# Examining the association between male circumcision and sexual function: evidence from a British probability survey

**DOI:** 10.1097/QAD.0000000000000745

**Published:** 2015-07-01

**Authors:** Virginia Homfray, Clare Tanton, Kirstin R. Mitchell, Robert F. Miller, Nigel Field, Wendy Macdowall, Kaye Wellings, Pam Sonnenberg, Anne M. Johnson, Catherine H. Mercer

**Affiliations:** a Centre for Sexual Health and HIV Research, Research Department of Infection and Population Health, University College London, London, UK; b Department of Social and Environmental Health Research, London School of Hygiene and Tropical Medicine, London, UK.

**Keywords:** circumcision, male, sexual dysfunction, sexual problem, survey

## Abstract

**Objective:**

Despite biological advantages of male circumcision in reducing HIV/sexually transmitted infection acquisition, concern is often expressed that it may reduce sexual enjoyment and function. We examine the association between circumcision and sexual function among sexually active men in Britain using data from Britain's third National Survey of Sexual Attitudes and Lifestyles (Natsal-3). Natsal-3 asked about circumcision and included a validated measure of sexual function, the Natsal-SF, which takes into account not only sexual difficulties but also the relationship context and overall level of satisfaction.

**Methods:**

A stratified probability survey of 6293 men and 8869 women aged 16–74 years, resident in Britain, undertaken 2010–2012, using computer-assisted face-to-face interviewing with computer-assisted self-interview for the more sensitive questions. Logistic regression was used to calculate odds ratios (ORs) to examine the association between reporting male circumcision and aspects of sexual function among sexually active men (*n* = 4816).

**Results:**

The prevalence of male circumcision in Britain was 20.7% [95% confidence interval (CI): 19.3–21.8]. There was no association between male circumcision and, being in the lowest quintile of scores for the Natsal-SF, an indicator of poorer sexual function (adjusted OR: 0.95, 95% CI: 0.76–1.18). Circumcised men were as likely as uncircumcised men to report the specific sexual difficulties asked about in Natsal-3, except that a larger proportion of circumcised men reported erectile difficulties. This association was of borderline statistical significance after adjusting for age and relationship status (adjusted OR: 1.27, 95% CI: 0.99–1.63).

**Conclusion:**

Data from a large, nationally representative British survey suggest that circumcision is not associated with men's overall sexual function at a population level.

## Introduction

The WHO states that there is compelling evidence to show that male circumcision reduces the risk of heterosexually acquired HIV and recommends that it is promoted as part of a comprehensive HIV prevention package [1]. However, there is concern that circumcision leads to reduced sexual satisfaction and a greater likelihood of experiencing sexual function problems, including erectile difficulties [2]. In 2013, a systematic review of male circumcision and its association with sexual function, sensitivity, and satisfaction found, from 36 articles, reporting data from 40 473 men, no difference between circumcised and uncircumcised men in terms of penile sensitivity, sexual arousal, sexual sensation, erectile function, premature ejaculation, ejaculatory latency, orgasm difficulties, sexual satisfaction, pleasure, or pain during penetration [3]. Included in this review were two large randomized controlled trials assessing adult circumcision as an intervention to prevent HIV, one in Kenya and the other in Uganda, which also assessed sexual pleasure and function in their 2-year follow-up and report conflicting results [4,5]. Although the Ugandan trial found no statistically significant difference in reported sexual satisfaction and pleasure between circumcised and uncircumcised men [5], in the Kenyan trial, circumcised participants reported greater penile sensitivity and ease of reaching orgasm [4].

In this article, we report analyses of data from the third British National Survey of Sexual Attitudes and Lifestyles (Natsal-3), a large national probability survey, which used a new, comprehensive, validated measure of sexual function, the Natsal-SF [6,7], allowing us to take into account not only sexual response but also the relationship context and men's overall level of satisfaction, to examine the association between circumcision and sexual function among sexually active men in Britain.

## Methods

Full details of the methods of the Natsal-3 have been reported elsewhere [8,9]. Briefly, a multistage, clustered, and stratified probability sample design was used. Altogether, 15 162 men and women aged 16–74 years and living in Britain were interviewed by computer-assisted personal interviews. Those reporting any sexual experience completed the more sensitive questions via computer-assisted self-interview (CASI). For this study, as in previous analyses of sexual function [10], we limit the denominator to sexually active men, defined as men reporting at least one sexual partner in the year prior to interview.

We used an overall measure of sexual function – the Natsal-SF, which is a 17-item validated measure developed for Natsal-3 [6,7]. It includes psychophysiological aspects of sexual function, including interest, enjoyment, anxiety, pain, arousal, timing of orgasm, lubrication (for women), and erectile function (for men), as well as considering the relationship context and self-appraisal of one's sex life. As in the previous publications [10,11], we consider those in the lowest quintile of the distribution of resulting scores for the Natsal-SF as having low sexual function relative to the rest of the population for the purpose of testing associations [12]. In addition to considering variations in sexual function by circumcision status according to this holistic measure, we also consider the association between circumcision and reporting the eight specific sexual ‘problems’ listed earlier, for at least 3 months in the past year, agreeing/agreeing strongly with the statement that *‘*I have avoided sex because of sexual difficulties, either my own or those of my partner’ (also one of the components of the Natsal-SF), and reporting ever taking any type of drug to assist sexual performance.

We used Stata, version 12 (StataCorp, College Station, Texas, USA) for all statistical analyses to account for the stratification, clustering, and weighting of the Natsal-3 sample. Data were weighted to match the resident British population according to the Census in terms of sex, age, and geographic region such that the weighted sample can be considered broadly representative of the British population [8,9].

Logistic regression was used to calculate odds ratios (ORs) to assess associations between circumcision status and the measures of sexual function mentioned previously. We used multivariable logistic regression to calculate adjusted OR (AOR) to adjust for the confounding effect of age and relationship status *a priori*. As numbers were small in some of the categories of ethnicity and religion wherein circumcision was more common, and combination of ‘non-white’ ethnicities and ‘non-Christian’ religions would have made too heterogeneous a group, we decided not to adjust for either ethnicity or religion after confirming that additional adjustment for ethnicity and religion affected the ORs only minimally. In addition, education did not confound the associations between circumcision and sexual function and so was not included in the adjusted model. The Natsal-3 study was approved by the Oxfordshire Research Ethics Committee A (reference: 09/H0604/27).

## Results

Of the 6293 men aged 16–74 years who participated in Natsal-3, 6117 (97.4%) reported whether they were circumcised, such that the prevalence of circumcision in the British male population is estimated as 20.7% (95% CI: 19.3–21.8). In total, 4816 of these 6117 men (78.7%) reported that they had at least one sexual partner in the year prior to interview and so were considered sexually active and included in the remaining analyses. Men who were sexually active were less likely to be circumcised (OR: 0.75, 95% CI: 0.62–0.90); however, after adjustment for age, there was no association between circumcision status and being sexually active (AOR: 0.84, 95% CI: 0.69–1.02). Table 1 shows the associations between circumcision and key sociodemographic factors in sexually active men. The prevalence of circumcision increased with age and was lower in those not currently living with a partner, although this association was reduced after adjusting for age. Men of ethnic minority backgrounds were more likely to report being circumcised than those of white ethnicity. Circumcision was strongly associated with Muslim religion [OR (vs. no religion): 29.82, 95% CI: 14.80–60.08] and was also higher in those reporting other religions, which included Jewish men [OR (vs. no religion): 2.51, 95% CI: 1.46–4.32].

Among sexually active men, there was no association between reporting having been circumcised and being categorized as in the lowest quintile of the Natsal-SF, an indicator of poorer sexual function overall (AOR: 0.95, 95% CI: 0.76–1.18; Table 2). Circumcised men were as likely as uncircumcised men to report all but one of the specific sexual function problems. The exception was reporting erectile difficulties, although, after adjusting for age and relationship status in multivariable analyses, this was of borderline statistical significance (AOR: 1.27, 95% CI: 0.99–1.63).

Similar proportions of sexually active circumcised and uncircumcised men – approximately one in 10 – agreed or agreed strongly that they had avoided sex in the past year because of sexual difficulties (either their own or their partner's), a finding that persisted when the denominator was expanded to include all men (data not shown). There was also no difference by circumcision status in the proportion of men who reported ever taking performance-enhancing drugs to assist their sexual performance.

## Discussion

Data from this large, national probability survey show that, among sexually active men in Britain, those who were circumcised were no more likely to have poor sexual function as measured by the Natsal-SF [6,7] than uncircumcised men. Although a slightly larger proportion of circumcised men reported erectile difficulties, this was of borderline statistical significance after adjusting for confounding sociodemographic characteristics.

Strengths of this study include the large sample size and the use of probability sampling, allowing us to present findings broadly representative of the British general population [8,9]. We rely on accurate reporting, which we acknowledge may be subject to reporting bias, although this is minimized by using CASI for the most sensitive questions including those on circumcision and sexual function. Of note, Natsal-3 did not ask men their age at circumcision, and although very few men in Britain are circumcised in adulthood (<1%; Dr Ruhin Karim, personal communication), we are not able to assess the immediate effect of circumcision, including upon sexual function.

As a survey addressing all aspects of sexual health and well-being, Natsal-3 did not have the scope to ask detailed questions about particular experiences of sexual function, for example, penile sensitivity. However, an important strength here is the holistic conceptualization of sexual function as captured by the Natsal-SF, allowing us to take into account not only sexual response, but also the relationship context and men's overall level of satisfaction. In addition, we did not have sufficient numbers of men reporting male partners within the past year to explore whether the associations between circumcision and sexual function varied by partner gender.

The lack of any observable association between circumcision and sexual function in this cross-sectional survey is in keeping with the conclusions from a large systematic review of male circumcision and its effect on men's sexual function, sensitivity, and satisfaction [2]. However, a Danish study concluded that it is the female partners of circumcised men who report greater dissatisfaction with their sex lives [13], although the Ugandan trial found no effect of male circumcision on female sexual satisfaction [14]. Although Natsal-3 did not ask about partners’ experience of sexual pleasure, we found no association between reporting circumcision and men's agreement with a statement regarding avoiding sex because of either their own or their partner's sexual difficulties. We recognize that this is an imperfect measure of partner's sexual pleasure and recommend that future studies address this. These limitations aside, we conclude that these data from a large, nationally representative British survey study provide further evidence that circumcision is not associated with male sexual function at a population level.

## Acknowledgements

The authors would like to thank the study participants, the team of interviewers from NatCen Social Research, and operations and computing staff from NatCen Social Research.

V.H., C.T., K.R.M., R.F.M., and C.H.M. conceived this article. V.H. wrote the first draft of the article, with further contributions from all other authors. V.H. did the statistical analysis, with support from C.T. and C.H.M. W.M., K.W., P.S., A.M.J., and C.H.M., initial applicants for Natsal-3, wrote the study protocol and obtained funding. C.T., K.R.M., N.F., W.M., K.W., P.S., A.M.J., and C.H.M. designed the Natsal-3 questionnaire, applied for ethics approval, and undertook piloting of the questionnaire. C.T. and C.H.M. managed data. All authors interpreted data, reviewed successive drafts, and approved the final version of the article.

Natsal-3 is a collaboration between University College London (London, UK), the London School of Hygiene and Tropical Medicine (London, UK), NatCen Social Research, Public Health England (formerly the Health Protection Agency), and the University of Manchester (Manchester, UK).

Sources of any support for the work in the form of grants, equipment, drugs, or any combination of these: N.F. is supported by a National Institute for Health Research Academic Clinical Lectureship.

Disclose funding received for this work from any of the following organizations: National Institutes of Health (NIH); Wellcome Trust; Howard Hughes Medical Institute (HHMI); and other(s): Natsal-3 was supported by grants from the Medical Research Council (G0701757) and the Wellcome Trust (084840), with contributions from the Economic and Social Research Council and Department of Health.

### Conflicts of interest

Disclaimers: A.M.J. has been a Governor of the Wellcome Trust since 2011. The other authors declare that they have no conflicts of interest.

## Figures and Tables

**Table 1:** Variation in the prevalence of circumcision among sexually active 16 to 74-year-old men by key sociodemographic factors.

Circumcised		OR		95% CI		*P* value		Age-adjusted OR		95% CI		*P* value		Denominator (unweighted, weighted)^a^^,^^b^	
%	95% CI
All	19.8	18.4–21.1							4816, 5955
Age (years)					<0.0001				1272, 929
16–24	12.8	10.8–15.1	1						1355, 1217
25–34	19.6	17.4–22.1	1.66	1.30–2.12					712, 1286
35–44	18.5	15.5–21.9	1.54	1.16–2.05					635, 1195
45–54	20.0	16.8–23.7	1.70	1.27–2.29					518, 860
55–64	21.1	17.6–25.1	1.82	1.35–2.45					324, 467
65–74	34.5	29.3–40.0	3.58	2.64–4.85					
Relationship status					<0.0001			0.0661	
Living with partner	21.5	19.8–23.3	1	–		1	–		2691, 4249
Steady relationship, not cohabiting	14.9	12.4–17.8	0.64	0.51–0.81		0.8	0.62–1.02		942, 754
No steady relationship, previously cohabited	15.9	12.5–20.1	0.69	0.51–0.93		0.74	0.55–0.99		447, 391
No steady relationship, never cohabited	15.5	12.6–18.9	0.67	0.51–0.88		0.95	0.71–1.27		725, 553
Academic qualifications^c^					0.0001			<0.0001	
No academic qualifications	18.7	15.6–22.3	1	–		1	–		708, 975
Academic qualifications typically gained at age 16	16.2	14.2–18.4	0.84	0.64–1.10		1.04	0.78–1.38		1638, 2021
Studying for/attained further academic qualifications	22.6	20.6–24.8	1.27	0.99–1.63		1.63	1.24–2.15		2283, 2791
Ethnic group					<0.0001			<0.0001	
White	16.3	15.1–17.7	1	–		1			4330, 5287
Mixed	35.6	24.5–48.4	2.83	1.67–4.80		3.70	2.15–6.37		89, 92
Asian/Asian British	49.3	40.8–57.9	4.98	3.48–7.11		5.63	3.90–8.13		199, 318
Black/Black British	53.6	43.2–63.6	5.91	3.85–9.06		6.75	4.23–10.75		146, 192
Other	34.0	19.9–51.6	2.63	1.28–5.41		3.33	1.59–6.98		45, 56
Religion					<0.0001			<0.0001	
None	16.2	14.6–17.9	1	–		1	–		2835, 3271
Christian	19.4	17.3–21.7	1.25	1.04–1.50		1.07	0.88–1.31		1708, 2297
Muslim	85.2	74.5–91.9	29.82	14.80–60.08		33.00	16.07–67.75		132, 193
Hindu	2.6	0.7–9.0	0.14	0.04–0.50		0.14	0.04–0.51		50, 85
Other	32.7	22.2–45.4	2.51	1.46–4.32		2.52	1.47–4.33		84, 99

CI, confidence interval; OR, odds ratio.

^a^Denominator defined as men aged 16–74 years reporting one or more sexual partner(s) in the year prior to interview for Britain's third National Survey of Sexual Attitudes and Lifestyles (Natsal-3).

^b^Unweighted, weighted denominators.

^c^Participants aged ≥17 years.

**Table 2:** Variation in the prevalence of different measures of sexual function according to circumcision status among sexually active men aged 16–74 years.

All men	By circumcision status		OR^d^ (95% CI)		*P* value		Adjusted OR^d^^,^^e^ (95% CI)		*P* value	
% (95% CI)	Circumcised % (95% CI)	Uncircumcised % (95% CI)
Denominator^a^^,^^b^	4816, 5955	866, 1176	3950, 4778	–	–	–	–
Lowest quintile for Natsal-SF measure^c^	19.9% (18.6–21.2)	20.2% (17.3–23.4)	19.8% (18.3–21.3)	1.03 (0.83–1.27)	0.814	0.95 (0.76–1.18)	0.625
Experienced the following for 3 or more months of the last year							
Lacked interest in having sex	15.0% (13.8–16.2)	14.5% (12.8–17.3)	15.1% (13.8–16.4)	0.95 (0.75–1.21)	0.695	0.93 (0.73–1.19)	0.585
Lacked enjoyment in sex	4.8% (4.2–5.5)	4.8% (3.5–6.7)	4.8% (4.1–5.6)	1.01 (0.69–1.48)	0.964	1.09 (0.74–1.60)	0.655
Felt anxious during sex	5.5% (4.8–6.2)	5.6% (4.2–7.4)	5.4% (4.7–6.3)	1.04 (0.72–1.49)	0.838	1.10 (0.76–1.59)	0.628
Felt physical pain as a result of sex	1.8% (1.4–2.3)	1.9% (1.1–3.3)	1.7% (1.4–2.3)	1.08 (0.58–2.00)	0.801	1.10 (0.60–2.03)	0.745
Felt no excitement or arousal during sex	3.2% (2.7–3.8)	3.5% (2.4–5.1)	3.1% (2.5–3.7)	1.15 (0.74–1.78)	0.527	1.24 (0.80–1.93)	0.337
Difficultly in reaching climax	9.2% (8.3–10.2)	9.3% (7.4–11.7)	9.2% (8.2–10.3)	1.01 (0.76–1.35)	0.929	1.03 (0.78–1.38)	0.814
Reached climax more quickly than you would like	14.9% (13.8–16.2)	15.3% (12.7–18.4)	14.8% (13.6–16.2)	1.04 (0.82–1.31)	0.747	1.09 (0.86–1.38)	0.484
Trouble getting or keeping an erection	13.0% (11.9–14.2)	16.7% (13.9–19.9)	12.1% (10.9–13.3)	1.46 (1.14–1.86)	0.002	1.27 (0.99–1.63)	0.062
Agree/agree strongly that avoided sex in past year due to sexual difficulties (own or partner's)	10.9% (10.0–12.0)	10.2% (8.0–12.8)	11.1% (10.0–12.3)	0.90 (0.68–1.20)	0.487	0.81 (0.61–1.08)	0.147
Ever taken performance enhancing drugs to assist sexual performance	13.0% (12.0–14.2)	14.2% (11.9–17.0)	12.8% (11.6–14.0)	1.14 (0.90–1.44)	0.286	1.04 (0.82–1.31)	0.776

CI, confidence interval; OR, odds ratio.

^a^Denominator defined as men aged 16–74 years reporting one or more sexual partner(s) in the year prior to interview for Britain's third National Survey of Sexual Attitudes and Lifestyles (Natsal-3).

^b^Unweighted, weighted denominators.

^c^See [6].

^d^Reference category is uncircumcised men.

^e^OR adjusted for age and relationship status.
